# Vitamin E Supplementation Enhances Lipid Oxidative Stability via Increasing Vitamin E Retention, Rather Than Gene Expression of MAPK-Nrf2 Signaling Pathway in Muscles of Broilers

**DOI:** 10.3390/foods10112555

**Published:** 2021-10-23

**Authors:** Lei Xu, Jing Wang, Haijun Zhang, Shugeng Wu, Hongyuan Yue, Xiaoli Wan, Haiming Yang, Zhiyue Wang, Guanghai Qi

**Affiliations:** 1 College of Animal Science and Technology, Yangzhou University, Yangzhou 225009, China; xlei@yzu.edu.cn (L.X.); wanxl@yzu.edu.cn (X.W.); hmyang@yzu.edu.cn (H.Y.); 2 Key Laboratory of Feed Biotechnology, Ministry of Agriculture, Feed Research Institute, Chinese Academy of Agricultural Sciences, Beijing 100081, China; wangjing@caas.cn (J.W.); zhanghaijun@caas.cn (H.Z.); wushugeng@caas.cn (S.W.); yuehongyuan@caas.cn (H.Y.); 3 Joint International Research Laboratory of Agriculture and Agri-Product Safety, Ministry of Education of China, Yangzhou University, Yangzhou 225009, China

**Keywords:** VE, MAPK, JNK, p38, Nrf2, GST, redox, lipid oxidation, meat, electrical stunning

## Abstract

Dietary vitamin E (VE) supplementation is a method to produce VE-enriched meat and improve meat lipid oxidative stability. We aimed to study the effect of the VE supplementation duration on meat lipid oxidative stability, VE retention, and antioxidant enzymes’ activity, and explore its relationship with the mitogen-activated protein kinases (MAPK)-nuclear factor-erythroid 2-related factor 2 (Nrf2) signaling pathway in broilers slaughtered after electrical stunning. A total of 240 male 18-day-old Arbor Acres Plus broilers were distributed to four treatments, with six replicates in each treatment, and ten broilers per replicate. Broilers were fed with a basal diet (no supplementation of VE) or VE diet (200 IU/kg VE, DL-α- tocopherol) for one (W1), two (W2), or three (W3) weeks before electrical stunning (130 mA, 60 Hz, for 1s) and slaughter. The VE retention was positively and linearly affected (*p* < 0.01) by the VE feeding duration at one to three weeks before slaughter, and negatively (all *p* < 0.01) related to the thiobarbituric acid reactive substance (TBARS) content in both breast and thigh muscles at d 0, d 2, and d 6 postmortem. The VE retention was negatively (*p* < 0.05) related to the gene expression of c-Jun N-terminal kinases 1 (JNK1) and 2 (JNK2), Nrf2 in breast muscles, and JNK1 and p38 MAPK in thigh muscles. In conclusion, dietary vitamin E supplementation at 200 IU/kg for three weeks before electrical stunning and slaughter improved lipid oxidative stability via increasing VE retention, rather than the regulation by gene expression of the MAPK-Nrf2 signaling pathway in skeletal muscles of broilers.

## 1. Introduction

Vitamin E (VE) supplementation in feed is one method to improve the antioxidant capacity and reduce lipid oxidation in the reproductive system, eggs, and offspring of chicken [1]. The VE has proved to be an effective antioxidant that protects unsaturated fatty acids against lipid peroxidation, and prevents membrane destabilization, cellular dysfunction [2], and the formation of undesirable sulfur compounds in raw meat [3]. Adding VE in feed enhances immunity, disease resistance, and the structural integrity of immune organs [4], and it improves meat quality via reducing lipid and protein oxidation in the early postmortem of animals [5,6]. Meat stability could be improved via feeding high concentrations of VE for two weeks before slaughter [7]. A supplement of VE in the forms of DL-α- or α- tocopherol acetate at 200 mg/kg feed for 2 to 3 weeks [8], 42 days [9], or 6 weeks [10] reduced thiobarbituric acid reactive substance (TBARS) in the broiler muscle and improved VE retention in the muscles. Electrical stunning is a widely used method in the world to make chickens unconscious and to relieve pain during slaughter. Electrical stunning could affect the production of reactive oxygen species (ROS) in muscle [11] and lipid oxidative stability during meat storage [11,12,13]. Previous studies of our team observed that electrical stunning with 130 mA, 60 Hz before slaughter decreased meat lipid peroxidation. We hypothesized that the supplementation of VE in feed for one to three weeks before electrical stunning (with 130 mA, 60 Hz) and slaughter could further improve meat lipid oxidative stability via increasing VE retention in skeletal muscles of broilers.

Nowadays, studies show that VE has both antioxidant and non-antioxidant effects through working on specific signal transduction enzymes, transcription factors, and membrane receptors at the molecular level [2]. The mitogen-activated protein kinases (MAPK)-nuclear factor-erythroid 2-related factor 2 (Nrf2) signaling pathway is reported to play an important role in antioxidant modulation in animals. Studies showed that VE has the biochemical activity to modulate signaling pathways (such as Nrf2), inflammatory molecules, apoptotic regulators, cytokines, kinases (e.g., MAPK), and antioxidant enzymes [14]. Tian et al. reported that VE could reduce fluorosis-induced oxidative stress and cell apoptosis through the suppression of c-Jun N-terminal kinases (JNK) and extracellular signal-regulated protein kinase (ERK) phosphorylation [15]. The VE has a therapeutic effect on lipid and glucose homeostasis through the activation of the Nrf2/carboxylesterase 1 (CES1) signaling pathway [16]. Vitamin E could protect rats from cadmium-induced sub-chronic liver injury via the activation of the Nrf2 pathway and the suppression of oxidative stress [17]. Vitamin E enhances the immunity and structural integrity of immune organs in grass carp associated with nuclear factor kappa-B (NF-κB), a target of rapamycin (TOR) and Nrf2 signaling [4]. The Impairment of the blood–testis barrier caused by di-(2-ethylhexyl) phthalate exposure could be alleviated by vitamin E and vitamin C through the inhibition of the oxidative stress-mediated p38 MAPK (p38) signaling pathway in rats [18]. VE also plays a critical role in the modulation of NF-κB, receptor activator of NF-κB/receptor activator of NF-κB ligand, MAPK, and oxidative stress signaling in bones [19]. Most previous studies focused on the effects of vitamin E on superoxide dismutase (SOD) and glutathione peroxidase (GPx) activities; however, the effects of vitamin E on the downstream targets of oxidative stress signaling need further study [19]. During our previous study, we observed that gas stunning with different CO_2_ concentrations [20] and electrical stunning with 130 mA, 60 Hz before slaughter affected gene transcriptions in the MAPK-Nrf2 signaling pathway in the muscle of broilers [12,21]. However, the changes in those antioxidant molecules played a limited role in lipid oxidation during storage [12,20,21], which was possibly due to a fast death of broilers after stunning (approximately 3 min). We hypothesized that adding VE to broiler diets for one to three weeks may improve lipid oxidative stability through increasing VE retention and the up-regulation of gene transcriptions in the MAPK-Nrf2 signaling pathway in the skeletal muscles of broilers.

We aimed to study the effect of the VE feeding duration on meat lipid oxidative stability, VE retention, and antioxidant enzyme activity, and explore its relationship with the MAPK-Nrf2 signaling pathway.

## 2. Materials and Methods

### 2.1. Birds and Treatment

Supplementation of VE at 200 IU/kg feed was proved to alleviate lipid oxidation in chicken eggs [1] and meat [8,9,10]. Thus, the concentration of 200 IU/kg feed was selected in this trial. A total of 240 male day-old Arbor Acres Plus broilers were reared in cages until 17-days old with a normal diet containing 20 IU/kg VE. The VE for treatment diet was provided from DL-α- tocopherol (50%) with 400 mg/kg in feed (i.e., 200 mg/kg VE in feed). Birds fasted for 8 h at the age of 17 days, and birds with similar bodyweight at 18-days old were randomly distributed to four treatments, with six replicates in each treatment, and ten broilers per replicate. Two diets were fed to broilers from 18 to 38 days. One was a basal diet with no supplementation of VE. The other was a treatment diet with VE supplementation at 200 IU/kg in feed. Birds from the control group were fed with the basal diet from 18 days to 38 days. Other birds were fed with the treatment diet for one (W1), two (W2), or three (W3) weeks before electrical stunning and slaughter. The diet composition and nutrient levels are shown in Table 1, and details of treatments are shown in Table 2. Birds had free access to feed and drinking water. The temperature, humidity, light, and immunization procedures were carried out according to the *Arbor Acres Broiler Management Guide* [22].

On day 38, one bird per replicate was selected (bodyweight 2.0 ± 0.2 kg). All of the selected birds were stunned with an alternating current of 130 mA, 60 Hz, for 1s in a water bath. Stunning parameters were set via an alternating current transformer (TDGC2-0.5 kVA, Hongbao Electric Co., Ltd., Zhejiang Yueqing, China). Stunning and slaughter were carried out according to our previous study [12]. The process of this study was approved by the Animal Care and Use Committee of the Yangzhou University with protocol NO. of YZUDWSY 2018-07-17.02.

### 2.2. Sampling

Blood was harvested in procoagulant tubes during bleeding from the jugular vein and carotid artery on the left side of the neck. Serum was collected after centrifuging at 1800× *g* at 4 °C for 10 min. Four pieces of meat (2.00 ± 0.20 g) were, respectively, taken from the left *Pectoralis Major* (breast muscle) and *Musculus Iliofibularis* (thigh muscle), stored at 4 (±0.25) °C for 0, 2, 4, 6 days, and then stored at −20 (±1) °C for measurement of TBARS and antioxidant capacity. Duplicate samples (0.20 ± 0.05 g) from the right side of *Pectoralis Major* and *Musculus Iliofibularis* were collected with sterilized scissors and tweezers and put into 0.50 mL DNase /RNase free plastic tubes, frozen in liquid nitrogen, and transferred to −80 °C for measuring of gene expression.

Approximately 20.0 (±3.0) g muscle sample was taken from the right side of *Pectoralis Major* and *Musculus Iliofibularis*, and stored at −80 °C for determination of VE. Two kilograms of feeds were taken during feed processing using a multipoint method, further reduced to 0.10 kg via quartering method, and stored at −80 °C for VE measurement.

### 2.3. Determination of α-Tocopherol (VE) in Feed and Muscles

15.0 g muscle was cut into small dices, crushed with a high-speed universal grinder (FW 100, Taisite Instrument Co., Ltd., Tianjin, China), dried with a freeze dryer (SCIENTZ-12N, Ningbo Xinzhi Freeze Drying Equipment Co., Ltd., Ningbo, China) at −70 °C for 72 h, and smashed for the second time with the same grinder. Feed samples (10.0 g) were smashed twice via the above grinder. Feed and muscle powder were sieved by 0.28 mm sieve, respectively, harvested in a sealing bag, and stored at −20 °C until analysis.

Feed was then measured via the method described in GB/T 17812-2008 [23]. Briefly, put feed powder (1.50 g) into an anaerobic tube, add with 6 mL ethanol, 1ml 10% L-ascorbic acid, and 2 mL KOH solution (KOH/water = 1:1, g/mL), fill with nitrogen, seal, mix using a vortex, give it a water bath (70 °C) for 30 min, and cool on ice. Transfer the saponification solution into a 50 mL tube with 2% NaCl (20 mL). Add 10 mL anhydrous ether, shake for 2 min, keep still for layering, and take the upper organic phase into a 50 mL centrifuge tube. Add anhydrous ether (5 mL) to extract α-tocopherol once again. Mix the upper organic phase (extracting solution) from the two extraction processes in a 50 mL centrifuge tube. Add 10 mL of water to wash ether, centrifuge at 2500× *g* at 4 °C for 10 min, take 5 mL of the upper organic phase, dry with nitrogen, dissolve with methanol to 1ml, and filter via membrane (0.45 μm) for further analysis. Extraction of α-tocopherol from muscle samples was similar to feed samples. The minor differences were that 0.50 g muscle was processed with 1mL 5% L-ascorbic acid and 15 mL anhydrous ether.

The α-tocopherol in feed and muscle extractions were measured via HPLC (E2695 of Waters, Milford, CT, USA) equipped with a chromatographic column (Zorbox sb-c18), with an inner diameter as 250 × 4.6 × 5 μm. The mobile phase was methanol/water at 98%:2%, with a flow rate of 1mL/min. The detection was carried out at 300 nm wavelength with an injection of 50 μL extraction. The standard curve and working curve were made according to the national standard (GB/T 17812-2008) [23]. Concentration of α-tocopherol was expressed as μg/g dry matter.

### 2.4. Lipid Oxidation and Antioxidant Capacity in Muscles and Serum

The TBARS was determined to represent lipid oxidation using a malondialdehyde (MDA) kit [12]. The contents of TBARS at d 0, d 2, d 4, and d 6 postmortem were recorded as TBARS_d0_, TBARS_d2_, TBARS_d4_, and TBARS_d6_. Protein contents and enzyme activities of SOD and glutathione S-transferase (GST) at d 0 and d 2 were measured according to our previous study [12,21] using protein kit (A045-2), total SOD kit (A001-3), and GST kit (A004) bought from Nanjing Jiancheng Bioengineering Institute (Nanjing, China). Measurement details were performed upon the instructions of the kits. For each variable, samples of all of the treatments were determined in one batch. The results of SOD and GST activity at d 0 and d 2 were recorded as SOD_d0_, SOD_d2_, GST_d0_, and GST_d2_, respectively. Serum uric acid was measured according to Xu et al. [21] using the uric acid kit (Shanghai Rongsheng Biopharmaceutical Co., Ltd., Shanghai, China). The TBARS, GST, and SOD of serum were measured using the same methods and commercial kits for muscles. Samples were determined in seven months.

### 2.5. Gene Expression

The gene expression of the MAPK-Nrf2 signaling pathway was measured using the method described in [12,20]. These molecules contained c-Jun N-terminal kinase 1 (JNK1), c-Jun N-terminal kinase 2 (JNK2), p38, Nrf2, the alpha 3 isozymes of glutathione S-transferase (GSTA3), the theta 1 isozymes of glutathione S-transferase (GSTT1), Cu/Zn-superoxide dismutase (SOD1), and Mn-superoxide dismutase (SOD2). The gene expression of β-actin was stable in the treatment of dietary VE; therefore, β-actin was chosen as a housekeeping gene in the present study. Primer sequences and annealing temperatures for real-time quantitive PCR were designed according to [20]. Results were normalized to β-actin mRNA levels based on the 2−ΔΔC^T^ method [24].

### 2.6. Statistical Analysis

Raw data were organized using Microsoft Excel 2016 of Microsoft Corp. (Beijing, China). Data were analyzed via the SPSS software (Ver. 20.0) for Windows from SPSS, Inc. (Chicago, IL, USA). Normal distribution of data was tested by Kolmogorov-Smirnov (K-S) tests. One-way ANOVA was applied to data analysis and means were separated by Duncan’s multiple range tests. Correlation between variables was analyzed with the bivariate Pearson’s correlation coefficients. Regression between variables was analyzed via linear regression. Results were expressed as means and SEM. The difference is significant if a *p*-value is lower than 0.05. Figures were created with OriginPro 8 SR1 (OriginLab Corporation, Northampton, MA, USA). Multiple figures were combined with Adobe Photoshop CC 2019 (Adobe Systems Inc., San Jose, CA, USA).

## 3. Results

### 3.1. VE Retention

**Figure 1:**
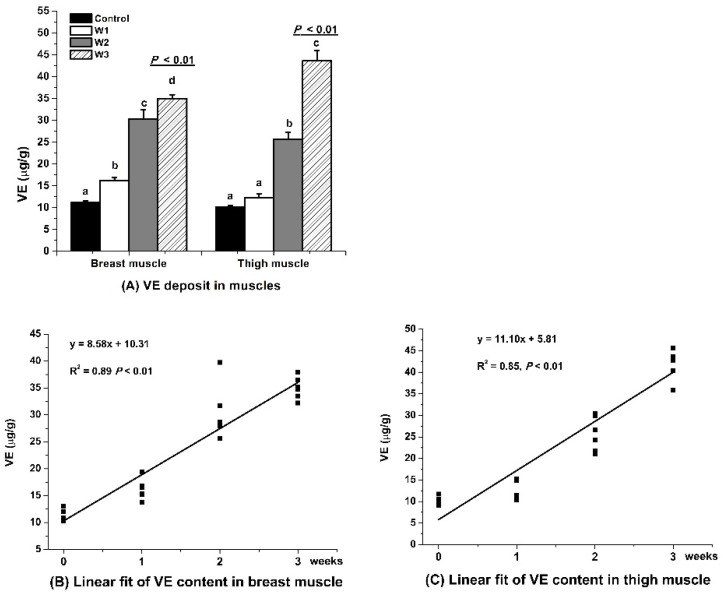
Effect of vitamin E (VE) feeding durations on VE retention in muscles of broilers slaughtered after electrical stunning. Control, W1, W2, and W3 represent the basal diet (without adding VE) or feed added with 200 IU/kg VE for one, two, or three weeks before the slaughter of broilers. ^a–d^ Means with no common superscripts within a group of columns differ significantly (all *p* < 0.01). Values are expressed based on the dry matter of muscles.

The VE content in both the breast and thigh muscles was greater (*p* < 0.01) in treatment W3 than W2 (*p* < 0.01), which is more (*p* < 0.01) than W1 and the control (*p* < 0.01, Figure 1A). The duration of feeding VE affected VE accumulation in both the breast (Figure 1B) and thigh muscles (Figure 1C) in both linear (*p* < 0.01) and quadratic (*p* < 0.01, figure not shown) manners during the first three weeks.

### 3.2. Lipid Oxidative Stability

**Figure 2:**
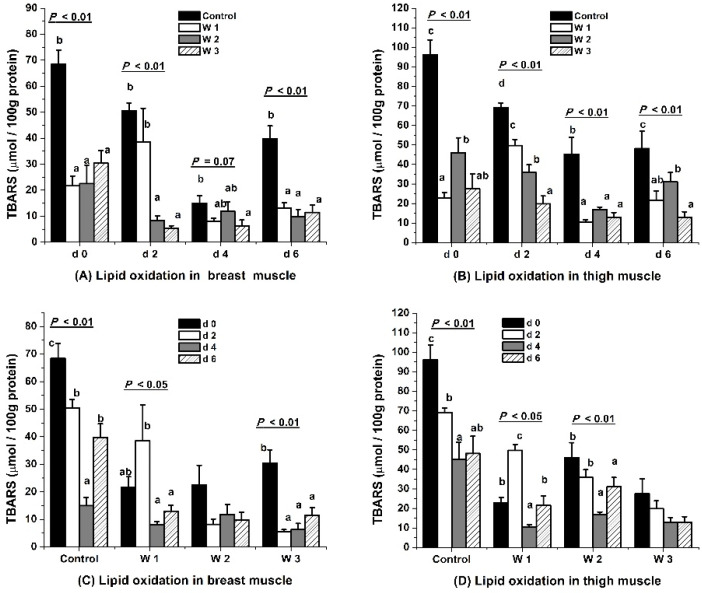
Effect of vitamin E (VE) feeding durations on lipid oxidative stability in muscles of broilers slaughtered after electrical stunning. Control, W1, W2, and W3 represent the basal diet (without adding VE) or feed added with 200 IU/kg VE for one, two, or three weeks before the slaughter of broilers. TBARS, thiobarbituric acid reactive substance (µmol malondialdehyde/100 g protein). ^a–d^ Means with no common superscripts within a group of columns differ significantly (*p* < 0.05).

Feeding VE for one to three weeks decreased TBARS at the day of slaughter (all *p* < 0.01) and during meat storage from d 2 to d 6 postmortem (all *p* < 0.01 except for d 4 in breast muscle, *p* = 0.07) in both the breast and thigh muscles of broilers as compared with the control (Figure 2A,B). The W3 consistently resulted in the lowest level (all *p* < 0.05) of TBARS in both breast and thigh muscles during six days of storage.

The TBARS remained at a relatively higher level and experienced an irregular increase or decrease during the storage of six days in the control and W1 in muscles (*p* < 0.05 or *p* < 0.01, Figure 2C,D). A supplementation of VE for two weeks resulted in a peak of TBARS on d 2 and a decrease in TBARS on d 4 and 6 in both breast (*p* < 0.05) and thigh (*p* < 0.01) muscles. Lipid oxidation was more stable in W2 (*p >* 0.05) and was the most stable in W3, where TBARS decreased from d 0 to d 2 (*p* < 0.01) and stayed at a low level between d 4 and d 6 (*p >* 0.05) in the muscles.

### 3.3. Antioxidant Capacity

**Figure 3:**
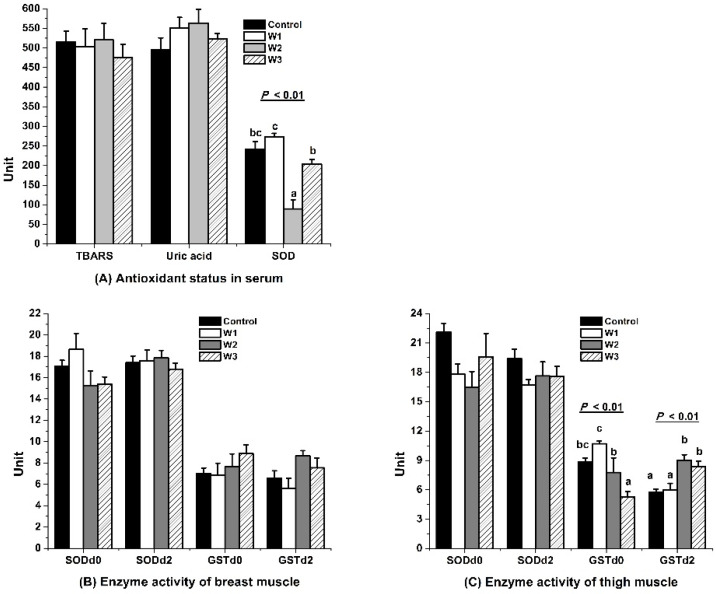
Effect of vitamin E (VE) feeding durations on antioxidant status in muscles of broilers slaughtered after electrical stunning. Control, W1, W2, and W3 represent the basal diet (without adding VE) or feed added with 200 IU/kg VE for one, two, or three weeks before the slaughter of broilers. TBARS, thiobarbituric acid reactive substance. SOD_d0_, SOD_d2_, GST_d0_, and GST_d2_, respectively, represent the activity of total superoxide dismutase and glutathione S-transferase at 0 d and 2 d postmortem; unit of TBARS, uric acid, SOD, and GST were µmol malondialdehyde/100 g protein, µmol/mg protein, U/mg protein, and U/mg protein. ^a–c^ Means with no common superscripts within a group of columns differ significantly (*p* < 0.05).

Feeding VE for two weeks decreased the activity of serum SOD and GST_d0_ in thigh muscles as compared to one week (*p* < 0.01, Figure 3A,C). Feeding VE for three weeks had a recovery of serum SOD and a further decrease in GST_d0_ in the thigh muscle. The activity of GST was higher in W2 and W3 than in the control and W1 at d 2. Serum TBARS, serum uric acid, GST_d0__–2_, and SOD_d0–2_ in the breast muscle, as well as SOD_d0__–2_ in the thigh muscle, remained at the same level among treatments (*p* > 0.05, Figure 3A–C).

### 3.4. MAPK-Nrf2 Signaling Pathway

**Figure 4:**
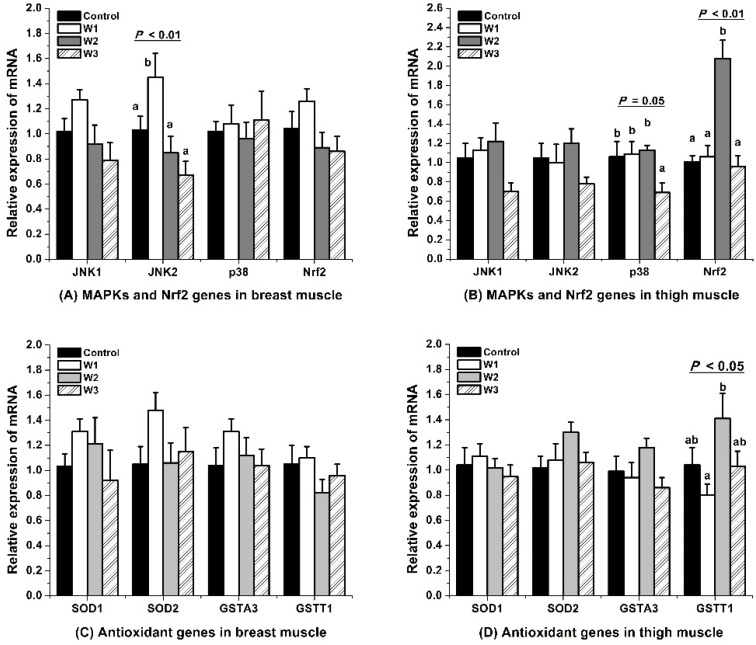
Effect of VE feeding durations on gene expressions of the mitogen-activated protein kinases (MAPK)-nuclear factor-erythroid 2-related factor 2 (Nrf2) signaling pathway in muscles of broilers slaughtered after electrical stunning. Control, W1, W2, and W3 represent the basal diet (without adding VE) or feed added with 200 IU/kg VE for one, two, or three weeks before the slaughter of broilers. JNK1, c-Jun N-terminal kinase 1; JNK2, c-Jun N-terminal kinase 2; p38, p38 mitogen-activated protein kinases, or p38 MAPK; Nrf2, nuclear factor-erythroid 2-related factor 2; GSTA3 and GSTT1, the alpha3 and theta 1 isozymes of glutathione S-transferase, respectively; SOD1 and SOD2, Cu/Zn- and Mn-superoxide dismutase, respectively. Results were normalized to β-actin mRNA levels. ^a–b^ Means with no common superscripts within a group of columns differ significantly (*p* < 0.05).

The JNK2 mRNA expression in breast muscle was up-regulated after feeding VE for one week, but recovered to the control level after two weeks (Figure 4A). The gene expression of Nrf2 in the thigh muscle was up-regulated by VE supplementation for two weeks, but recovered to the control level at the third week (Figure 4B). Feeding VE for three weeks suppressed (*p* = 0.05) mRNA expression of p38 in thigh muscle. The duration of VE feeding had no influence (*p* > 0.05) on other genes of the MAPK-Nrf2 pathway in the breast or thigh muscle compared to the control group.

### 3.5. Correlations among Variables

The feeding duration strongly and positively affected (all *p* < 0.01) VE retention in both breast and thigh muscles (Table 3). In the breast muscle, VE retention was positively related to the activity of GST_d2_ (*p* < 0.05), but negatively related to TBARS at d 0, d 2, and d 6 (all *p* < 0.01), and gene expressions of JNK1 (*p* < 0.05), JNK2 (*p* < 0.01), and Nrf2 (*p* < 0.05). In the thigh muscle, VE retention was positively related to the activity of GST_d2_ (*p* < 0.01), but negatively related to GST_d0_ (*p* < 0.01), TBARS from d 0 to d 6 postmortem (*p* < 0.05 or <0.01), and the gene expression of JNK1 and p38 in thigh muscles (*p* < 0.05).

The feeding duration of VE was negatively related to the TBARS content in both breast (*p* < 0.01) and thigh muscles (*p* < 0.05 or < 0.01) from d 0 to d 6, excepted for the TBARS_d4_ of the breast muscle (*p* > 0.05, Table 4). The TBARS_d0_ and TBARS_d4_ levels were positively (both *p* < 0.05) correlated with SOD_d2_ activity in the thigh muscle. The TBARS_d2_ was negatively correlated with GST_d0_ (*p* < 0.05) and GST_d2_ (*p* < 0.01) in the breast muscle and GST_d2_ (*p* < 0.01) in the thigh muscle. The TBARS_d2_ was positively related to the mRNA expressions of JNK2 (*p* < 0.01) and Nrf2 (*p* < 0.05) in the breast muscle, and GST_d0_ activity (*p* < 0.01) in the thigh muscle. The TBARS_d6_ was positively (*p* < 0.05) related to mRNA expressions of JNK1 and GSTA3 in the thigh muscle. However, TBARS at the day of slaughter was not related to any mRNA expression in the genes of the MAPK-Nrf2 pathway (all *p* > 0.05).

## 4. Discussion

### 4.1. VE Retention

The duration of VE feeding increased VE retention in both breast and thigh muscles in a time-dependent, linear, and quadratic manner in the first three weeks. Consistent with our study, the feeding duration of VE directly affected the content of VE deposited in the muscle cell membranes [25]. A gradual increase in the VE content was observed in both the breast and thigh muscle from one to three weeks before slaughter [8]. Increasing the VE content enhanced the VE content in a linear style in the breast meat regardless of VE sources in the diet [26]. Dietary supplementation with vitamin E increased the concentrations of VE in the breast muscle [27], some other tissues, and plasma [28]. VE is a potential agent to protect humans from cancer, diabetes, lipid disorder, radiation damage, and allergic, cardiovascular, bone, eye, liver, inflammatory, and neurological disease [14,17,19]. The present study indicated that a supplementation of VE at 200 IU/kg in feed for three weeks before slaughter may be an effective way to produce natural VE in chicken meat as human’s functional food.

### 4.2. Lipid Oxidative Stability

Feeding VE for one to three weeks, especially for three weeks, decreased TBARS in both the breast and thigh muscles of broilers at the day of slaughter and during six days’ cold storage. Similar to our study, a VE supplement of 200 mg/kg feed for 2 to 3 weeks [8] and six weeks [9,10] reduced lipid oxidation in both breast and thigh meat. However, a supplement of VE for a short period (12 days) did not affect meat lipid oxidation [29]. Lipid oxidation was decreased by dietary E in chickens exposed to heat stress [6] or ochratoxin A-induced stress [27]. This antioxidant effect could be caused by VE alone [26,28,30] or by VE with butylated hydroxyanisole [5] or selenium [3].

In the present study, lipid oxidative stability was improved in muscles from broiler fed VE at 200 IU/kg feed for two (thigh muscle) or three weeks (breast and thigh muscles) during six days of storage. Consistent with our study, a higher level of dietary VE in broiler diets resulted in better lipid oxidation stability in meat [31]. In the present study, the feeding duration of VE positively related to VE retention, and the latter was negatively related to the TBARS content in breast and thigh muscles during the storage for six days. The TBARS decreased as the storage time was prolonged in the breast meat of W2 and W3, for the first four days of the control, and the last five days of W1. A possible reason might be that TBARS was further metabolized to be smaller molecules (such as acetate, methylglyoxal, acetaldehyde, CO_2_ and H_2_O, and D-lactate,) or to have a chemical reaction with proteins and DNA in order to form bigger adducts [32]. In the control group, TBARS underwent an increase on d 6 in raw breast meat. This increase was advanced to d 2 in the breast meat of W1, but no increase in TBARS was observed in W2 and W3. Increasing VE in the diet improves the content of monounsaturated fatty acids (MUFA) and polyunsaturated fatty acids (PUFA) in breast and thigh meat [30,33]. These unsaturated fatty acids are sensitive to lipid oxidation and might be one reason for the higher TBARS on d 6 of the control and d 2 of W1, where VE accumulation was not enough to prevent lipid peroxidation. In addition, the transcriptional expression of oxidative sensitive JNK2 was up-regulated in W1. These data indicated that supplementing VE for one week might stimulate oxidative stress in the muscle, which exacerbated lipid oxidation during the first two days of storage. Enzyme activities of both antioxidases and oxidases are reduced by the proteolysis, crosslink, or breakdown of protein as the storage time increases [34,35]. However, a non-enzymatic system is still efficient in driving lipid peroxidation without the involvement of oxidases [36]. Vitamin E is present in the membrane components of the cell in order to prevent peroxide formation [37]. A higher VE retention in muscles could enhance the non-enzyme antioxidant capacity of meat, which may explain why TBARS was not increased in W2 or W3 during storage. Thus, a greater VE retention in muscles and its non-enzyme antioxidant effect might be the reason why chickens fed VE for a longer time had better lipid oxidative stability.

### 4.3. Antioxidant Capacity

The SOD plays a vital role in the ability of VE to prevent oxidative damage [38]. In the present study, the activity of serum SOD was increased by feeding VE for two weeks, but recovered in the third week. VE selectively increased SOD in some tissues [39]. The activity of SOD was enhanced by VE in carp injected with bacteria [4], and in chicken fed naturally contaminated diets with ochratoxin A [27]. However, the SOD activity in the blood was reduced in mice gavage with VE [40], or not affected in chickens fed with different VE isomers and doses [28] or in rat testis [15]. Data indicated that the serum SOD might be spared by a higher antioxidant capacity induced by a greater VE accumulation in the body at W2. However, a new balance of serum SOD was reached after feeding VE for three weeks. The SOD_d2_ activity was positively correlated with TBARS at d 0 and d 4, indicating that an increase in TBARS at the day of slaughter may stimulate an increase in SOD_d2_; however, when the broilers were dead, the residual SOD activity in the muscle in a cold environment (4 °C) was not enough to cope with the lipid oxidation on d 4.

The GST is one class of phase II detoxifying enzymes that helps to maintain cell integrity, oxidative balance, and prevention against DNA damage via catalyzing the conjugation of glutathione (GSH) to various electrophilic substrates [41]. Feeding VE for two and three weeks reduced the GST in the thigh muscle on the day of slaughter compared with the control and feeding VE for one week. However, VE retention positively affected the activity of GST_d2_ in breast and thigh muscles. The reason might be that dietary VE results in higher values of the antioxidant capacity of lipid-soluble compounds [28]. Dietary VE in feed increases non-enzymatic antioxidants in the muscle [27]. Similarly, VE prevented a fluoride-induced decrease in the GSH level [15] and alleviated oxidative damage via enhancing the activity of GPx and glutathione reductase (GR) in carp injected with bacteria [4]. This indicated that the increase in VE retention reduced the need for antioxidant-related enzymes when chickens were alive. When muscles were stored at a low temperature, the VE retention in the tissue may have superiority over the antioxidant enzyme in fighting against lipid oxidation, preventing the GST from depletion, which leads to a higher activity of GST_d2_.

### 4.4. MAPK-Nrf2 Signaling Pathway

MAPK-Nrf2 is a potential way to regulate oxidative damage and lipid peroxidation in cells [42,43]. The JNK1, JNK2, and p38 are members of MAPKs, and they phosphorylate transcription factors (e.g., Nrf2) either bound or unbound to DNA [44]. Via the phosphorylation and activation of Nrf2 by p38 MAPK, Nrf2 translocate to the nucleus, up-regulating phase II enzymes (e.g., GST) to fight against oxidative injury [45]. The GSTA and GSTT gene families mainly catalyze the conjugation of various electrophiles produced from oxidative stress with reduced GSH in cytosolic [46]. Stunning broilers with 130 mA, 60 Hz may reduce the oxidative stress and improve the lipid oxidative stability of the muscle during storage from 1 to 9 d [12]. Although gene expressions in the MAPK-Nrf2 pathway were affected by gas stunning [20] or electrical stunning [12,21], lipid oxidative stability during storage was not correlated with mRNA expression. In the present study, all of the groups were stunned with 130 mA, 60 Hz. Thus the difference in mRNA expression among groups could be attributed to the effect of dietary VE.

Feeding the broiler with VE for a short period (one to two weeks) up-regulated the gene expression of JNK2 and Nrf2, whereas feeding VE for a longer time (three weeks) down-regulated these mRNAs to the control level. The VE could up-regulate the Nrf2 mRNA level to ameliorated oxidative stress in carps facing oxidative damage induced by bacteria [4], in mice with nonalcoholic fatty liver disease induced by fructose [16], or in rats with sub-chronic liver injury induced by cadmium [17]. Although the over-generation of ROS leads to cellular peroxidation and the pathogenesis of multiple diseases, a proper physiological level of ROS is necessary for cellular functions [19]. A reduced generation of ROS below physiological levels may impair their action as second messengers, leading to reduced adipocyte differentiation, lipid transport, and adipogenesis [40]. Therefore, long-term and high doses of VE may bring side effects. For instance, mice were insulin-resistant after receiving VE gavage for 14 weeks [40]. Consistent with our study, Alcala et al. [40] also observed a reduction in the Nrf2 mRNA expression followed by increases in the VE deposit in retroperitoneal white adipose tissue of mice gavage with VE. Four weeks’ treatment with VE decreased the RNA expression of JNK1 and reduced c-jun phosphorylation in the hypercholesterolemia-induced atherosclerosis model of rabbits [47]. The data of our study indicated that the gene expression of the JNK-Nrf2 signaling pathway might be up-regulated by VE supplementation for a short time, but may be down-regulated by adding VE for three weeks in order to maintain a balance in broilers.

The VE retention was negatively related to the TBARS and gene expressions of JNK1, JNK2, and Nrf2 in the breast muscle and JNK1 and p38 in the thigh muscle. Consistently, the mRNA expressions of JNK2 and Nrf2 were positively related to TBARS_d2_ in the breast muscle, and JNK1 and GSTA3 were positively related to TBARS_d6_ in the thigh muscle. Lipid oxidation was consistently controlled by VE through the suppression of molecules in the p38-Nrf2-ARE signaling pathway in rat testis challenged by di-(2-ethylhexyl) phthalate [18]. The JNK and p38K were observed to be activated by a high-fat diet but to be inhibited by the addition of walnuts (a source of VE) in feed [48]. In other studies, JNK and Nrf2 were also down-regulated by VE addition in the conditions of various challenges [4,15,47,49]. One reason may be that a longer VE feeding duration increased the VE retention, leading to a reduction in lipid oxidation in the muscle tissue, which reduced the need for the MAPK-Nrf2-induced antioxidant system. Another reason might be that the activation of MAPKs by ROS is also an indicator of stress, which leads to apoptosis [50]. Preventing the over-activation of this pathway or regulating these molecules to the control level is good for the physical balance in broilers. In agreement with this deduction, cell apoptosis was suppressed by VE via the down-regulation of MAPK (ERK and JNK) pathways [15]. The activation of NF-κB and interleukin secretion were suppressed by VE in endothelial cells that were cultured exposed to lipopolysaccharide (LPS)/peptidoglycan G [47]. The VE may protect the body from lipid disorders, inflammatory diseases, and other diseases [14,17,19], via up-regulating the MAPK-Nrf2-related genes in the first two weeks, but down-regulating them to a normal level on the third week in the present study.

Lipid oxidation on the day of slaughter was not related to any mRNA expression in the genes of the MAPK-Nrf2 pathway. This indicated that this pathway may have a limited effect on the protection of muscular lipid oxidation when broilers are alive. During storage, TBARS_d2_ or TBARS_d6_ were positively related to the mRNA expressions of JNK2, Nrf2, JNK1, and GSTA3, indicating MAPKs and Nrf2 genes are sensitive to oxidative imbalance and tried to protect muscles via up-regulating the mRNA expressions. However, the antioxidant effect of the MAPK-Nrf2-related enzymes was trivial when muscles were stored at a low temperature [12,20,21]. Traber and Atkinson suggest that almost all of the biological activities of VE result from its antioxidant function to protect polyunsaturated fatty acids and membrane qualities [51]. The addition of VE in feed can increase the concentrations of non-enzymatic antioxidants, e.g., vitamin C, retinol, total tocopherols, and the VE equivalent in broiler muscles [27]. VE, as one of the non-enzyme antioxidants, may have a more stable function than the MAPK-Nrf2 pathway mediated antioxidant enzymes in protecting muscles from lipid oxidation during cold storage.

## 5. Conclusions

The VE supplementation duration enhanced the VE retention in muscles in a linear and quadratic manner in broilers three weeks before electrical stunning and slaughter. This is an effective way to produce VE-enriched meat and improve meat lipid oxidative stability via feeding broiler with 200 IU/kg VE in feed for three weeks. Data from the present study did not support our hypothesis that VE might improve meat lipid oxidative stability through the up-regulation of gene expressions in the MAPK-Nrf2 signaling pathway. Although a long-term supplementation of VE in feed suppressed the gene expression of the MAPK-Nrf2 signaling pathway, it enhanced lipid oxidative stability at the day of slaughter and during cold storage via increasing the non-enzyme antioxidant effect through VE retention in the skeletal muscles of broilers slaughtered after electrical stunning.

## Figures and Tables

**Table 1:** Composition and nutrient levels of basal diets (air-dry basis, %).

Ingredients	Feed Composition	
	d 0–17	d 18–38
Corn	63.00	55.90
Soybean	31.74	37.93
Soybean oil	1.82	2.23
Limestone	1.36	1.19
Dicalcium phosphate	1.30	1.87
Lysine hydrochloride	0.05	0.05
DL-methionine	0.07	0.17
Salt	0.35	0.35
Choline	0.23	0.23
Vitamin premix ^1^	0.02	0.02
Mineral premix ^2^	0.20	0.20
Nutrient levels ^3^		
Metabolizable energy, MJ/kg	12.22	12.39
Crude protein, %	22.60	20.30
Lysine, %	1.24	1.08
Methionine, %	0.48	0.36
Methionine + Cystine, %	0.82	0.68
Calcium, %	0.94	0.86
Non-phytate phosphorus, %	0.43	0.34

^1^ Vitamin premix supplied following vitamins for each kg of feed: vitamin A, 1,400 IU; D3, 185IU; E, 20 IU during d 0–17 and 0 IU in basal diet during d 18–38; K3, 1.0 mg; B1, 2.04 mg; B2, 8.13 mg; B6, 3.39 mg; B12, 1.0 mg; biotin, 0.25 mg; pantothenic acid, 10.2 mg; folic acid, 0.57 mg; niacin, 32.67 mg. ^2^ Mineral premix supplied following minerals for each kg of feed: Mn, 200 mg; Zn,151 mg; Cu, 11 mg; Fe 150 mg; Se, 0.35 mg; I, 1.0 mg. ^3^ Nutrient levels are calculated values.

**Table 2:** Treatment details.

Treatments ^1^	Dietary VE Content (IU/kg)				VE Feeding Duration	Electrical Stunning (Alternative Current)
	d 0–17	d 18–24	d 25–31	d 32–38		
Control	20	0	0	0	None	130 mA, 50 Hz, 1 s
W1	20	0	0	200	One week	130 mA, 50 Hz, 1 s
W2	20	0	200	200	Two weeks	130 mA, 50 Hz, 1 s
W3	20	200	200	200	Three weeks	130 mA, 50 Hz, 1 s

^1^ Control, W1, W2, and W3 represent the basal diet (without adding DL-a-tocopherol), or a feed added with 200 IU/kg DL-a-tocopherol for one, two, or three weeks, respectively, before the slaughter of broilers. VE content in the diet of d 0–17, the basal diet of d 18–38, and the treatment diet of d 18–38, respectively, contained vitamin E of 22.99, 1.53, and 217.74 IU/kg in feed (measured values).

**Table 3:** Correlation coefficients between vitamin E (VE) retention and other variables in broiler muscles.

Items ^1^	Variables ^2^	VE Retention	
		Breast Muscle	Thigh Muscle
VE	Feeding duration	0.94 **	0.92 **
	TBARS_d0_	−0.53 **	−0.42 *
TBARS	TBARS_d2_	−0.72 **	−0.85 **
	TBARS_d4_	−0.33	−0.44 *
	TBARS_d6_	−0.60 **	−0.51 *
	SOD_d0_	−0.04	−0.14
Enzyme activity	SDD_d2_	−0.07	−0.07
	GST_d0_	0.22	−0.72 **
	GST_d2_	0.40 *	0.58 **
MAPKs genes	JNK1	−0.49 *	−0.43 *
	JNK2	−0.52 **	−0.23
	p38	0.01	−0.44 *
Nrf2 gene	Nrf2	−0.45 *	0.03
Antioxidant genes	SOD1	−0.10	−0.25
	SOD2	−0.16	0.06
	GSTA3	−0.21	−0.14
	GSTT1	−0.30	0.11

^1^ MAPK, mitogen-activated protein kinase; Nrf2, nuclear factor-erythroid 2-related factor 2; ^2^ TBARS_d0_, TBARS_d2_, TBARS_d4_, and TBARS_d6_, thiobarbituric acid reactive substance at 0, 2, 4, 6 d postmortem. SOD_d0_, SOD_d2_, GST_d0_, and GST_d2_, respectively, represent total superoxide dismutase and glutathione S-transferase at 0 and 2 d postmortem; JNK1, c-Jun N-terminal kinase 1; JNK2, c-Jun N-terminal kinase 2; p38, p38 mitogen-activated protein kinases, or p38 MAPK; GSTA3 and GSTT1 alpha3 and theta 1 isozymes of glutathione S-transferase, respectively; SOD1 and SOD2, Cu/Zn- and Mn-superoxide dismutase, respectively. * and **, respectively, indicate a correlation significance at the 0.05 level or 0.01 level (*n* = 24, two-tailed).

**Table 4:** Correlation coefficients between TBARS and antioxidant variables in broiler muscles.

Tissues	Items ^1^	Variables ^2^	TBARS			
			TBARS_d0_	TBARS_d2_	TBARS_d4_	TBARS_d6_
Breast muscle	VE	Feeding duration	−0.56 **	−0.76 **	−0.37	−0.68 **
	Enzyme activity	SOD_d0_	−0.15	−0.07	−0.19	0.24
		SDD_d2_	−0.07	−0.13	−0.09	0.17
		GST_d0_	−0.19	−0.42 *	−0.26	−0.14
		GST_d2_	−0.21	−0.51 **	0.12	−0.20
	MAPKs genes	JNK1	0.02	0.29	0.35	0.09
		JNK2	0.01	0.65 **	0.31	0.06
		p38	0.02	0.10	0.33	0.10
	Nrf2 gene	Nrf2	0.11	0.45 *	0.29	0.10
	Antioxidant genes	SOD1	−0.15	0.07	0.17	0.05
		SOD2	−0.06	0.15	0.15	−0.14
		GSTA3	0.03	−0.06	0.25	−0.09
		GSTT1	0.30	0.37	0.21	0.38
Thigh muscle	VE	Feeding duration	−0.62 **	−0.92 **	−0.59 **	−0.57 **
	Enzyme activity	SOD_d0_	0.33	0.22	0.23	0.09
		SDD_d2_	0.47 *	0.24	0.44 *	0.05
		GST_d0_	0.22	0.51 **	0.09	0.27
		GST_d2_	−0.16	−0.78 **	−0.34	−0.27
		JNK1	0.11	0.25	0.22	0.49 *
	MAPKs genes	JNK2	0.11	0.12	0.34	0.28
		p38	0.27	0.28	0.12	0.30
		Nrf2	0.01	−0.22	−0.20	0.06
	Nrf2 gene	SOD1	0.03	0.05	0.36	0.30
	Antioxidant genes	SOD2	0.02	−0.34	−0.13	0.05
		GSTA3	0.14	0.01	0.31	0.45 *
		GSTT1	0.27	−0.18	0.15	0.25

^1^ MAPK, mitogen-activated protein kinase; Nrf2, nuclear factor-erythroid 2-related factor 2; ^2^ SOD_d0_, SOD_d2_, GST_d0_, and GST_d2_, respectively, represent total superoxide dismutase and glutathione S-transferase at 0 d and 2 d postmortem; JNK1, c-Jun N-terminal kinase 1; JNK2, c-Jun N-terminal kinase 2; p38, p38 mitogen-activated protein kinases, or p38 MAPK; GSTA3, alpha3 isozymes of glutathione S-transferase; GSTT1, the theta 1 isozymes of glutathione S-transferase; SOD1, Cu/Zn-superoxide dismutase; SOD2, Mn-superoxide dismutase. * and **, respectively, indicate a correlation significance at the 0.05 level or 0.01 level (*n* = 24, 2-tailed).

## Data Availability

The data in this study are available on reasonable request from the corresponding author.
